# Genetic and Clinical Heterogeneity of Polish Patients with Congenital Stationary Night Blindness (CSNB)

**DOI:** 10.3390/ijms27114855

**Published:** 2026-05-28

**Authors:** Lukasz Kuszel, Anna Wawrocka, Joanna Walczak-Sztulpa, Anna Skorczyk-Werner, Maciej R. Krawczynski

**Affiliations:** 1 Department of Medical Genetics, Poznan University of Medical Sciences, 8 Rokietnicka Str., 60-806 Poznan, Poland; aniawawrocka@ump.edu.pl (A.W.); jsztulpa@ump.edu.pl (J.W.-S.); askorczyk@ump.edu.pl (A.S.-W.); mrkrawcz@ump.edu.pl (M.R.K.); 2 GENESIS Diagnostics, 77A Dabrowskiego Str., 60-529 Poznan, Poland

**Keywords:** congenital stationary night blindness, CSNB, diagnostic NGS panel, whole-exome sequencing

## Abstract

Congenital stationary night blindness (CSNB) is a rare, genetically and clinically heterogeneous group of non-progressive inherited retinal diseases characterized by night blindness, myopia, nystagmus, and decreased visual acuity, for which comprehensive genetic characterization remains essential to enable accurate diagnosis and future gene therapy development. In this study, we performed a clinical and genetic analysis of twenty-one Polish families diagnosed with CSNB using next-generation sequencing (NGS)-based targeted gene panels and, in one case, whole-exome sequencing (WES), complemented by Sanger sequencing for variant validation and segregation analysis. Pathogenic variants were identified in six genes: *GPR179* and *NYX* were the most frequently affected (six families each), followed by *CACNA1F* (three families), *GRM6*, *TRPM1*, and *SLC24A1* (two families each). The complete Schubert–Bornschein form predominated in our cohort, in contrast to previous reports indicating higher prevalence of the incomplete form. Notably, ten previously unreported variants were identified in *CACNA1F*, *GRM6*, and *NYX*, expanding the known mutational spectrum of CSNB. Certain variants appear enriched in the Polish population. These findings underscore the value of NGS-based approaches for precise molecular diagnosis of CSNB and contribute to the broader understanding of its genetic architecture.

## 1. Introduction

Congenital stationary night blindness (CSNB) belongs to a group of rare non-syndromic, non-progressive inherited retinal diseases. It is a genetically and clinically heterogeneous disorder. CSNB is characterized primarily by night blindness, myopia, nystagmus, and decreased visual acuity. Affected individuals may also exhibit strabismus and fundus abnormalities. CSNB symptoms show a childhood onset. Clinical features are associated with defective signal processing from photoreceptor cells to bipolar cells in the retina. CSNB is associated with various forms of inheritance, including autosomal dominant (AD), autosomal recessive (AR), and the most common X-linked (XL) form of CSNB.

An accurate diagnosis of this disorder requires a fundoscopic examination and non-invasive full-field electroretinography (ffERG), which is crucial for determining the CSNB subtype. CSNB can be classified into two groups: CSNB with a normal fundus image and CSNB with fundus abnormalities [1,2,3].

Fundus albipunctatus (OMIM #136880) and Oguchi disease (OMIM #258100 type 1 and OMIM #613411 type 2) belong to CSNB with fundus defects, and both are inherited in an autosomal recessive manner.

CSNB with no fundus anomalies can be diagnosed as Schubert–Bornschein type and Riggs type. Moreover, the Schubert–Bornschein type can be further divided into two subtypes: complete CSNB (cCSNB, CSNB1) and incomplete CSNB (icCSNB, CSNB2) [3,4].

The Schubert–Bornschein type is the most common form of CSNB and can be inherited in an AR or X-linked manner.

The Riggs type can be associated with an AD or AR mode of inheritance. The Riggs type of CSNB is characterized by dysfunction in rod photoreceptors [2,5].

To date, 18 genes are known to cause CSNB without fundus abnormalities [6].

X-linked CSNB is caused by pathogenic variants in the *CACNA1F* gene (OMIM*300071) and the *NYX* gene (OMIM*300278), known to be related to icCSNB type and cCSNB type, respectively. The phenotype is associated with optic disc tilt, optic nerve atrophy, and morning glory syndrome. Nystagmus can also be present.

So far, three genes have been associated with the autosomal dominant form of CSNB, namely *GNAT1* (OMIM*610444), *PDE6B* (OMIM*163500), and *RHO* (OMIM*300071). Patients with this form of inheritance have a normal fundus with no nystagmus.

An autosomal recessive mode of inheritance of this disorder is caused by thirteen genes. These include *CABP4* (OMIM*608965), *GNAT1* (OMIM*616389), *GUY2D* (OMIM*618555), *GNB3* (OMIM*617024), *GPR179* (OMIM*614515), *GRK1* (OMIM*613411), *GRM6* (OMIM*257270), *LRIT3* (OMIM*615058), *RDH5* (OMIM*601617), *SAG* (OMIM*181031), *SLC24A1* (OMIM*613830), and *TRPM1* (OMIM*603576) [4,6,7].

In recent years, research projects focusing on gene therapies for retinal genetic disorders have made significant progress. CSNB is a non-progressive disease, making it an interesting candidate for gene therapy treatment. From another site, finding a therapeutic solution for CSNB is challenging due to the narrow therapeutic window, as most patients with CSNB exhibit poor vision in infancy [2]. Recently, several animal models have been developed for CSNB, including those of mice, horses, dogs, rats, zebrafish, and flies. Nevertheless, therapies for CSNB have not been discovered so far. Designing specific gene therapies for inherited eye disorders remains challenging, as it involves a significant amount of time for the entire process, encompassing genetic diagnosis, designing the therapy, drug production, and preclinical testing [3,7,8]. Therefore, genetic diagnostics and understanding the molecular pathogenesis of CSNB-related genes will provide opportunities for developing gene therapy and improving the quality of life for affected individuals.

Here, we report a clinical and genetic analysis of a cohort of twenty-one Polish families diagnosed with CSNB. These include nine families with X-linked CSNB and twelve families with the autosomal recessive form of CSNB. This is the first study on a Polish group of patients with congenital stationary night blindness. So far, data concerning Central and Eastern European populations have been limited. Our findings show that the prevalence of genes related to CSNB in our population differs from populations associated with the high frequency of consanguinity [1].

## 2. Results

### 2.1. Clinical Features

A cohort of twenty-one patients with a confirmed diagnosis of congenital stationary night blindness and twenty-nine family members was enrolled in the study. Patients exhibit the classical manifestations of CSNB. Almost all patients develop high myopia (no data available for patients P48, P52, and P55). The pathological alterations primarily affect the rod photoreceptors, which are responsible for scotopic vision. In contrast, cone function is typically normal or only slightly reduced. Night blindness was identified in 16 patients, whereas 2 patients did not report impaired scotopic vision (P36, P54). Reduced visual acuity was observed in the majority of patients. Normal visual acuity was documented only in patients P23 and P55. The detailed ophthalmological findings of the patients are summarized in Table 1.

### 2.2. Molecular Results

Comprehensive molecular investigations employing NGS-based targeted gene panels and whole-exome sequencing (WES) revealed variants in six genes implicated in this disorder. Ten of the identified variants have not been previously described in the literature in association with CSNB. In the studies performed, the most prevalent gene defects were variants in *GPR179* (CSNB1E; 28.6%) and *NYX* (CSNB1A; 28.6%), followed by *CACNA1F* (CSNB2A; 14.3%), *GRM6* (CSNB1B; 9.5%), *SLC24A1* (CSNB1D; 9.5%), and *TRPM1* (CSNB1C; 9.5%). The identified genetic variants are presented and summarized in Table 2.

**Figure 1:**
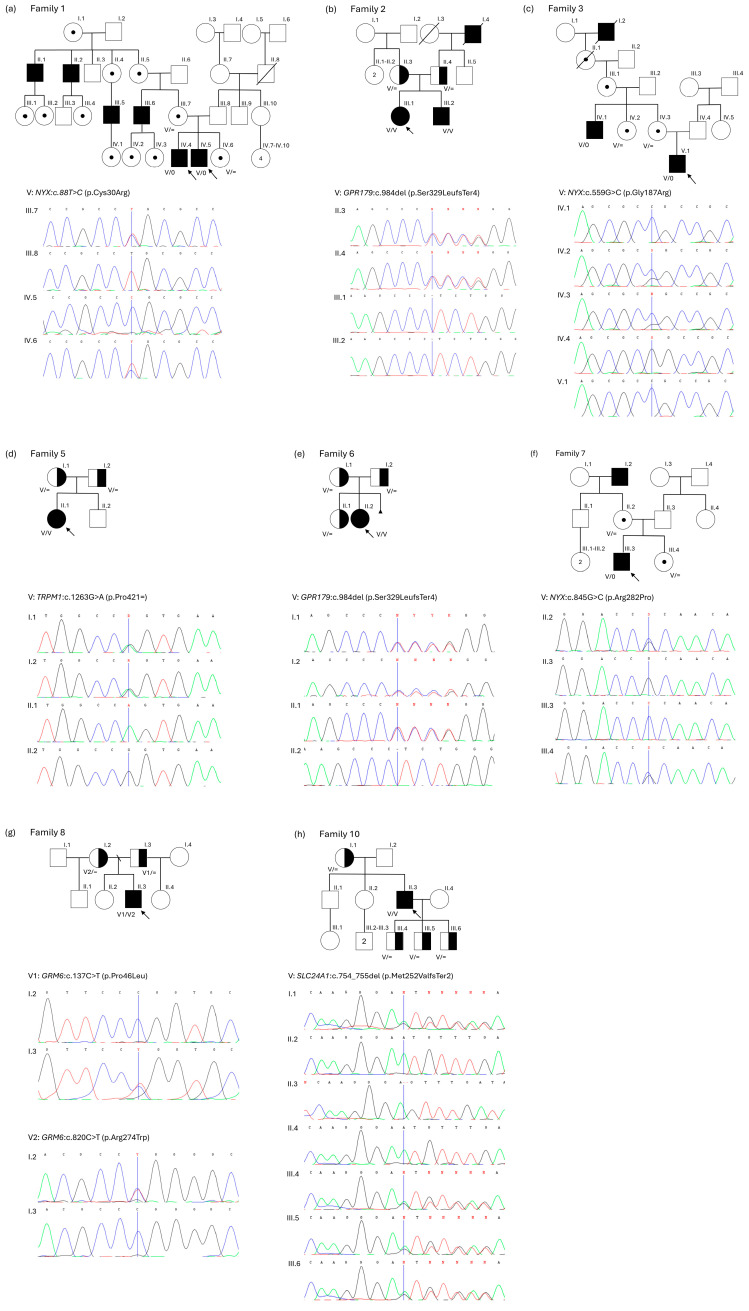
The pedigrees and segregation of the variants associated with the disease in patients’ families. (**a**)—family 1; (**b**)—family 2; (**c**)—family 3; (**d**)—family 5; (**e**)—family 6; (**f**)—family 7; (**g**)—family 8; (**h**)—family 10. The identified variants are shown below the pedigrees. Symbols used in pedigrees: square—male; circle—female; empty symbol—unaffected individual; filled/black symbol—affected by the trait or disease; half-shaded symbol—carrier (for recessive traits); dot inside a circle—female carrier of an X-linked trait; diagonal slash through symbol—deceased; the arrow indicates the proband; small triangle—miscarriage; diagonal slash through partner line—divorce; generations are labeled with Roman numerals and individuals within a generation are numbered left to right; V/V—homozygote; V/=—heterozygote; V/0—hemizygote.

Subsequently, invitations to participate in the segregation analysis of the identified variants were sent to 21 probands’ families. Among those invited, eight families agreed to participate in the study. In these families, pedigree analyses were carried out alongside molecular characterization of the detected variants. The outcomes of these investigations are summarized in Figure 1.

## 3. Discussion

Congenital stationary night blindness comprises a group of disorders characterized by marked heterogeneity in inheritance patterns, causative genes, and clinical and electrophysiological manifestations. Establishing a precise diagnosis based exclusively on a standard ophthalmological examination, in the absence of a family history of the disease, is frequently exceedingly challenging. Recent advances in high-throughput sequencing technologies have enabled accurate and highly precise diagnosis of CSNB.

NGS analyses enabled unequivocal confirmation of the CSNB type in 21 examined patients. The complete form of the Schubert–Bornschein type (cCSNB) was identified in 15 patients (P1, P7, P10, P15, P20, P23, P27, P31, P46, P47, P49, P50, P51, P52, P55), incomplete form (iCSNB) was identified in 3 patients (P36, P54, P57), and the Riggs type was confirmed in two patients (P39 and P48). According to the literature, the incomplete Schubert–Bornschein form is reported to be the most common, which is inconsistent with our findings in this patient’s cohort. In our study, the cCSNB form clearly predominated. The incomplete form is characterized by less pronounced impairment of night vision. In fact, approximately half of the affected patients do not report complaints related to night vision [7]. In our cohort, patients with the iCSNB P36 and P54 did not experience night vision difficulties, whereas patient P57 did. The complete Schubert–Bornschein form is typically associated with night blindness, reduced visual acuity to approximately 0.5, and moderate to high myopia. In the studied group, patients reported symptoms of night blindness, and a marked reduction in visual acuity was observed in the majority of cases. Exceptions were patients P15, P23, and P55, in whom visual acuity remained within the normal range. Strabismus and nystagmus are also frequently observed in patients with cCSNB type, with nystagmus tending to diminish with age [4]. Among the 15 patients included in the study with cCSNB, strabismus was identified in 5 cases (P7, P20, P27, P47, P49) and nystagmus in 6 cases (P7, P10, P27, P31, P47, P50); both conditions co-occurred in 3 patients (P7, P27, and P47). In the Riggs form, night blindness is observed, whereas visual acuity is typically unimpaired [9]. Both patients, P39 and P48, reported difficulties with night vision. In patient P48, visual acuity was close to normal, whereas patient P39 exhibited a markedly reduced visual acuity (Table 1).

The highest prevalence was observed for *GPR179* and *NYX* (six families each), whereas alterations in *CACNA1F* were identified in three families, and alterations in *GRM6*, *TRPM1*, and *SLC24A1* were identified in two families each.

The most frequently identified variant in our cohort was p.Ser329LeufsTer4 in the *GPR179* gene associated with the autosomal recessive complete form of congenital stationary night blindness (Schubert–Bornschein type; CSNB1E). This change has been found in all six *GPR179*-affected individuals. Four out of six patients were homozygous, and two were compound heterozygotes for this gene alteration. This variant in a homozygous state has also been found in one out of three CSNB Polish patients described by Durajczyk et al. [6], indicating that the frequency of p.Ser329LeufsTer4 change might be relatively higher in the Polish population as compared to the general population. These findings are within the range of allele frequency in the Polgenom database estimated at 0.4%, and the allele frequency described in the general population in the GnomAD database, reported as 0.03493%. However, large studies described by AItalbishi et al., including 161 CSNB patients from 76 families, showed that defects in the *TRPM1* gene are the most identified in the Palestinian and Israeli populations with autosomal recessive CSNB [10].

Similar results have been reported by Sundaramurthy et al., who identified the *TRPM1* gene as the most prevalent gene alteration in the Indian cohort [11]. The *TRPM1* gene was also the most frequent cause of CSNB in the article described by Huang et al. [4]. *TRPM1* variants have also been identified in two out of six CSNB families reported by Kim et al. [2]. Additionally, genetic analysis in a cohort of CSNB patients from Saudi Arabia showed the recessive variants in *TRPM1* and *CABP4* accounted for the majority of affected individuals. These findings might be associated with the high frequency of consanguinity in the populations analyzed [1].

Interestingly, recent large studies reported by Katta et al. performed in 122 CSNB individuals (107 CSNB families) revealed the presence of variants in 50 families in the *CACNA1F* gene, *NYX* variants in 22 families, *TRPM1* defects in 19 families, *GRM6* changes in 13 families, *GPR179* in 2 families, and only 1 patient has been found to have the *CABP4* gene variant [7]. Other reports also indicate the frequent involvement of *CACNA1F*, *NYX*, and *GRM6* genes in the pathogenesis of CSNB [4,11].

Another variant, p.Met252ValfsTer2 in *SLC24A1*, was homozygous in two families with CSNB in our cohort. The frequency of this alteration in the general population, based on GnomAD, is estimated at 0.0176% and in the Polgenom database at 0.79%, suggesting that this change is more common in the Polish population.

In the present study, we identified 10 previously unreported variants associated with CSNB in three genes: *CACNA1F*, *GRM6*, and *NYX*, each encoding a protein essential for signal transmission from photoreceptors to bipolar cells in the retina. These findings expand the known mutational spectrum of CSNB and reinforce its genetic heterogeneity.

Three novel variants were detected in *CACNA1F*. Two of these, c.4588+1G>A and c.3942+1G>C, are located at the donor splice site of their respective introns, disrupting the canonical splice donor consensus sequence. According to ACMG criteria, variants at this position constitute very strong evidence of pathogenicity. Although splice-site mutations in *CACNA1F* have been previously reported, these specific variants have not been described in association with CSNB. The third *CACNA1F* variant, c.1018C>T (p.Gln340Ter), is a nonsense substitution that converts a glutamine codon to a premature termination codon at position 340 of the Ca_v_1.4 protein (voltage-gated calcium channel). The p.Gln340Ter variant truncates the protein within the first repeat domain (domain I), eliminating the vast majority of the transmembrane segments (S1–S6 of domains II–IV), the voltage-sensing apparatus, the pore-forming regions, and the extensive C-terminal regulatory domain [12,13,14].

Two previously unreported variants were identified in *GRM6*. The first, c.152_174dup (p.Gln59AlafsTer10), is a duplication within the N-terminal extracellular ligand-binding domain that results in a frameshift and premature termination after ten aberrant amino acids. This region is critical for glutamate binding, and disease-associated mutations in the ligand-binding domain have been shown to abolish proper protein trafficking to the cell surface, with mutant mGluR6 being retained in the endoplasmic reticulum [12,15]. This variant is cataloged in the gnomAD population database and has been assigned an rs identifier, indicating that it has been observed in the general population. However, to date, c.152_174dup has not been reported in association with CSNB or any other retinal disorder in the published literature. Its presence in gnomAD does not preclude pathogenicity, as autosomal recessive CSNB requires biallelic mutations, and heterozygous carriers are expected to be phenotypically normal. The second *GRM6* variant, c.445_453del (p.Val149_Ala151del), is an in-frame deletion removing three amino acids within the ligand-binding domain. The variant also has an rs id but has not been associated with CSNB so far. Although such deletions are less frequently reported than frameshift or missense variants, removal of residues from the glutamate-binding region is likely to alter receptor conformation and impair ligand binding or protein folding. Previous studies have demonstrated that even single amino acid changes in the ligand-binding domain can abolish mGluR6 surface localization [15].

Finally, five novel missense variants were identified in the *NYX* gene: c.559G>C (p.Gly187Arg), c.845G>C (p.Arg282Pro), c.565A>T (p.Ile189Phe), c.788G>T (p.Gly263Val), and c.446T>C (p.Leu149Pro). All identified variants are located within the leucine-rich repeat (LRR) region of the protein. The LRR domain, situated in the extracellular space, forms a curved solenoid structure that mediates key protein–protein interactions and contributes to the assembly of a macromolecular signaling complex required for ON-bipolar cell function. In particular, this domain enables direct interactions with TRPM1 and mGluR6, which are essential components of the ON-bipolar signaling pathway [16,17,18]. Variants affecting conserved residues within the LRR region are therefore likely to disrupt the domain’s structural integrity and impair its interactions with downstream signaling partners.

Collectively, these findings expand the allelic spectrum of CSNB and support the need for comprehensive genetic screening of all known CSNB-associated genes to enable accurate molecular diagnosis. However, due to cohort size and limited access for segregation analysis in families described (eight out of twenty-one) further investigations and observations are necessary to increase our knowledge concerning CSNB. Only in this way the visual prognosis, mode of inheritance, and optimal management can be unequivocally determined.

## 4. Materials and Methods

### 4.1. Clinical Examination

Twenty-one patients affected with CSNB and twenty-nine family members were recruited from twenty-one families. Both the patients and all family members were of Polish origin. In the study group, eight cases were familial and thirteen sporadic.

The patients were previously referred for ophthalmologic examination, including measurement of central visual acuity (18 patients), full-field electroretinography (ERG; 18 patients), and optical coherence tomography (OCT; 15 patients). Patients were provided with genetic counseling in conjunction with a comprehensive medical and family history analysis.

The study was conducted in accordance with the ethical principles outlined in the Declaration of Helsinki and its subsequent amendments and was approved by the local Bioethics Committee at the Poznan University of Medical Sciences (approval no. 485/24). Written informed consent was obtained from the patients and, for patients under 18 years, from their parents.

### 4.2. Molecular Analysis

Genomic DNA used for molecular NGS testing was extracted from peripheral blood samples collected from probands. Blood was drawn using a vacuum collection system into EDTA-containing tubes, and DNA was subsequently isolated from peripheral blood leukocytes with the MagCore^®^ HF16 Automated Nucleic Acid Extractor and the Genomic DNA Large Volume Whole Blood Kit (RBC Bioscience Corp., New Taipei, Taiwan).

Molecular testing was conducted in each proband using next-generation sequencing (NGS) with a targeted gene panel for inherited ocular disorders. In one case, whole-exome sequencing (WES) was additionally performed. NGS analyses were outsourced to external laboratories, including Genomed (Warsaw, Poland) and Asper Biogene (Tartu, Estonia). The technical specifications of these procedures are summarized and included in the Supplementary Materials (Table S1). Bioinformatic analyses were performed according to the protocol described by Matczynska et al. [19]. Molecular analyses were performed between 2017 and 2025, during which the gene panel underwent several modifications, leading to differences in certain parameters. The NGS gene panels differed mainly in the total number of analyzed genes, reflecting the ongoing development and expansion of diagnostic panels over time. However, all genes known to be associated with congenital stationary night blindness (CSNB) were included in each version of the panel; therefore, these modifications did not affect the detection of variants in established CSNB-related genes. Due to the large number of analyzed genes and the evolving composition of the panels, detailed gene lists are available from the authors upon reasonable request.

The decision to use diagnostic whole-exome sequencing (WES) in one patient was based on the patient’s young age (2 years at the time of testing), progressive myopia, and the presence of additional clinical features, including mild developmental delay and a cavernous hemangioma involving the left side of the face. Owing to the patient’s young age, several ophthalmological procedures could not be performed, which precluded a complete ophthalmic evaluation. WES analysis was conducted according to the routine diagnostic pipeline, with particular emphasis placed on genes included in ophthalmic gene panels as well as genes selected on the basis of the extraocular manifestations, thereby enabling a broader diagnostic assessment.

Validation of identified variants in probands and segregation analyses in selected families was performed using Sanger sequencing of PCR-amplified products. For these procedures, genomic DNA was obtained from buccal swabs following the manufacturer’s protocol (NucleoSpin Tissue Kit; Macherey-Nagel, Düren, Germany). PCR primers were designed with Primer3Plus software (version 3.3.0, available online). Primer sequences are available upon request. For variant classification according to the American College of Medical Genetics (ACMG) guidelines, three online tools were used: Varsome (varsome.com; version 13.15.1.0; access date: 16 April 2026) [20], Franklin by Genoos (franklin.genoox.com; version 93; access date: 16 April 2026), and GeneBe (genebe.net; beta version; access date: 16 April 2026) [21].

## Figures and Tables

**Table 1:** Clinical symptoms of the patients with CSNB.

Family/Patient ID	Current Age/Gender	CSNB Type	Ophthalmic Symptoms							
			Night Blindness	Family History	ERG Results	Myopia	BCVA RE/LE (VIS?)	VF Restriction	OCT	Additional Ophthalmological Symptoms
**F1/P1**	20/M	CSNB1A	+	+	Scotopic—extinguished Photopic—normal	−15.0D	0.4–0.5	Irregular visual field defects	Thinning of the retina	-
**F2/P7**	13/F	CSNB1E	+	+	Scotopic—decreased Photopic—reduced amplitudes	−7.0D +astigmatism	0.3–0.25	ND	Thinning of the retina	Bilateral strabismus that resolved spontaneously; color vision impairment; mild nystagmus
**F3/P10**	3/M	CSNB1A	+	+	Scotopic—extinguished Photopic—normal	−3.0D +astigmatism (−1.5D)	ND	ND	ND	Horizontal nystagmus
**F4/P15**	7/F	CSNB1B	+	−	Scotopic—extinguished	−9.0D +astigmatism	0.9–1.0	ND	Normal	-
**F5/P20**	6/F	CSNB1C	+	−	Scotopic—extinguished Photopic—reduced amplitudes	−7.0D	0.3–0.4	ND	Normal	Oblique strabismus
**F6/P23**	17/F	CSNB1E	+	−	Scotopic—extinguished	Myopic astigmatism	1.0	ND	ND	-
**F7/P27**	9/M	CSNB1A	+	+	Scotopic—extinguished	−8.0D	0.4	ND	Normal	Nystagmus, strabismus
**F8/P31**	9/M	CSNB1B	+	−	Scotopic—extinguished Photopic—reduced amplitudes	from −3.0D to −4.0D + myopic astigmatism	0.2–0.3	Narrowing of the visual field	Thinning of the retina	Infantile nystagmus
**F9/P36**	10/M	CSNB2A	−	−	Scotopic—extinguished	−5.5D +myopic astigmatism (−2.5D)	0.5	-	Normal	-
**F10/P39**	39/M	CSNB1D	+	−	ND	Mixed astigmatism	0.2–0.3	Narrowing of the visual field to approx. 15 degrees	Perifoveal photoreceptor atrophy	-
**F11/P46**	3/M	CSNB1E	ND	−	ND	−5.0D	ND	ND	Thinning of the retina	-
**F12/P47**	9/M	CSNB1A	+	+	Scotopic—extinguished Photopic—normal	Myopic astigmatism	0.8–0.25	ND	Normal	Mild nystagmus and divergent strabismus
**F13/P48**	47/M	CSNB1D	+	+	Scotopic—decreased Photopic—decreased	ND	0.7–1.0	Narrowing of the visual field to approx. 20 degrees	Perifoveal photoreceptor atrophy RE—normal macula, LE—lamellar macular hole	-
**F14/P49**	16/M	CSNB1A	+	−	Scotopic—extinguished Photopic—decreased	−6.5D	0.6–0.8	ND	Normal	Convergent strabismus
**F15/P50**	8/M	CSNB1E	ND	−	Scotopic—extinguished Photopic—within the lower limits of normal	Myopic astigmatism	0.6–0.8	ND	ND	Horizontal nystagmus
**F16/P51**	51/M	CSNB1C	+	−	Scotopic—decreased Photopic—normal	Myopic astigmatism	0.2–0.8	Irregular visual field defects	Photoreceptor atrophy	-
**F17/P52**	14/M	CSNB1E	+	-	Scotopic—extinguished Photopic—reduced amplitudes	Slight myopia	0.6–0.8	ND	Normal	-
**F18/P54**	11/M	CSNB2A	−	+	Scotopic—extinguished with the b-wave decreased Photopic -with the b-wave decreased Abnormal oscillatory potentials	−9.0D + myopic astigmatism (−2.0D)	0.4–0.3	ND	Normal	-
**F19/P55**	50/F	CSNB1E	+	−	Scotopic—decreased Photopic—disturbed morphology without electronegative recording	ND	1.0	Irregular visual field defects	Borderline of normal	-
**F20/P56**	7/M	CSNB1A	+	+	Scotopic—extinguished Photopic—reduced	Myopia	0.6–0.7	Normal	ND	-
**F21/P57**	7/M	CSNB2A	+	−	Scotopic—extinguished Photopic—reduced amplitudes	Myopic astigmatism	0.4–0.5	-	Abnormal profile of the fovea fundus	-

BCVA—best-corrected visual acuity; RE—right eye; LE—left eye; M—male; F—female; VF- visual field; ND—no data; +present, −absent.

**Table 2:** DNA variants identified by NGS in patients.

Patient/Family	Gene	Transcript	Variant Classification		Inheritance	ACMG Classification			ClinVar	Molecular Method of Searching the Variants	GnomAD v4.1.0 Total Allele Frequency
			Nucleotide	Protein		Varsome	Franklin	GeneBe			
**F9/P36**	*CACNA1F*	NM_001256789.3	**c.4588+1G>A**	**Splice donor variant**	XLR	LP	LP	P	Not reported	NGS panel	-
**F18/P54**	*CACNA1F*	NM_001256789.3	**c.3942+1G>C**	**Splice donor variant**	XLR	LP	LP	VUS	Not reported	NGS panel	-
**F21/P57**	*CACNA1F*	NM_001256789.3	**c.1018C>T**	**p.Gln340Ter**	XLR	LP	LP	P	Not reported	NGS panel	-
**F2/P7**	*GPR179*	NM_001004334.4	c.984del rs770066665	p.Ser329LeufsTer4	AR	P	P	P	P/LP	NGS panel	0.0003493
**F6/P23**	*GPR179*	NM_001004334.4	c.984del rs770066665	p.Ser329LeufsTer4	AR	P	P	P	P/LP	NGS panel	0.0003493
**F11/P46**	*GPR179*	NM_001004334.4	c.984del rs770066665	p.Ser329LeufsTer4	AR	P	P	P	P/LP	WES	0.0003493
**F15/P50**	*GPR179*	NM_001004334.4	c.984del rs770066665 c.1368del rs1435030978	p.Ser329LeufsTer4 p.Phe456LeufsTer30	AR	P P	P P	P P	P/LP P	NGS panel NGS panel	0.0003493 0.00001921
**F17/P52**	*GPR179*	NM_001004334.4	c.984del rs770066665 c.1141C>T rs749683775	p.Ser329LeufsTer4 p.Arg381Trp	AR	P VUS	P VUS	P VUS	P/LP VUS	NGS panel NGS panel	0.0003493 0.00001555
**F19/P55**	*GPR179*	NM_001004334.4	c.984del rs770066665	p.Ser329LeufsTer4	AR	P	P	P	P/LP	NGS panel	0.0003493
**F4/P15**	*GRM6*	NM_000843.4	**c.152_174dup** rs1760743459 **c.445_453del** rs2480407301	**p.Gln59AlafsTer10** **p.Val149_Ala151del**	AR	P VUS	P VUS	P VUS	P Not reported	NGS panel NGS panel	0.000001466 0.000001373
**F8/P31**	*GRM6*	NM_000843.4	c.137C>T rs62638197 c.820C>T rs577125911	p.Pro46Leu p.Arg274Trp	AR	LP VUS	LP VUS	P VUS	LP VUS	NGS panel NGS panel	0.0001538 0.000008676
**F1/P1**	*NYX*	NM_001378477.3	c.88T>C rs1292184180	p.Cys30Arg	XLR	VUS	VUS	LP	VUS	NGS panel	0.000
**F3/P10**	*NYX*	NM_001378477.3	**c.559G>C**	**p.Gly187Arg**	XLR	VUS	VUS	VUS	Not reported	NGS panel	-
**F7/P27**	*NYX*	NM_001378477.3	**c.845G>C**	**p.Arg282Pro**	XLR	VUS	VUS	LP	Not reported	NGS panel	-
**F12/P47**	*NYX*	NM_001378477.3	**c.565A>T**	**p.Ile189Phe**	XLR	VUS	VUS	VUS	Not reported	NGS panel	-
**F14/P49**	*NYX*	NM_022567.2	**c.788G>T**	**p.Gly263Val**	XLR	VUS	VUS	VUS	Not reported	NGS panel	-
**F20/P56**	*NYX*	NM_001378477.3	**c.446T>C**	**p.Leu149Pro**	XLR	VUS	VUS	VUS	Not reported	NGS panel	-
**F10/P39**	*SLC24A1*	NM_004727.3	c.754_755del rs777989874	p.Met252ValfsTer2	AR	P	P	P	P/LP	NGS panel	0.0001760
**F13/P48**	*SLC24A1*	NM_004727.3	c.754_755del rs777989874	p.Met252ValfsTer2	AR	P	P	P	P/LP	NGS panel	0.0001760
**F5/P20**	*TRPM1*	NM_001252024.2	c.1263G>A rs768701595	p.Pro421=	AR	LP	LP	VUS	Conflicting classifications	NGS panel	0.000008056
**F16/P51**	*TRPM1*	NM_001252020.1	c.332A>G rs200514769 c.3397C>T rs779371614	p.Tyr111Cys p.Arg1133 *	AR	LP LP	P LP	VUS P	Conflicting classifications Not reported	NGS panel NGS panel	0.0001887 0.000004008

XLR—X-linked recessive, AR—autosomal recessive, LP—likely pathogenic, P—pathogenic, VUS—uncertain significance, NGS—next generation sequencing, WES—whole-exome sequencing. American College of Medical Genetics (ACMG) classification was obtained through the Varsome, Franklin, and GeneBe online available tools. New variants identified in this study are bolded. * stop codon.

## Data Availability

The original contributions presented in this study are included in the article/Supplementary Material. Further inquiries can be directed to the corresponding author.

## Supplementary Materials

The following supporting information can be downloaded at: https://www.mdpi.com/article/10.3390/ijms27114855/s1.
