# Supplementary material for: Genetic and Clinical Heterogeneity of Polish Patients with Congenital Stationary Night Blindness (CSNB)
Source: Int J Mol Sci. 2026 May 28;27(11):4855. doi: 10.3390/ijms27114855 (PMC13256437; doi:10.3390/ijms27114855)
Supplement: Supplementary file 1 [file ijms-27-04855-s001.zip › ijms-4295851-supplementary.pdf]

Table S1. Technical details of the NGS technique.

Patient/Family	Laboratory name	NGS Kit	Laboratory equipment for reading the sequence	Test code	Number of genes analyzed	Coverage for the sequences of the studied regions of the analyzed genes		Coverage for mitochondrial genome sequences	
						Average coverage	Detailed coverage	Average coverage	Detailed coverage
**F1/P1**	Genomed	Twist Bioscience Custom Panel (Twist Bioscence)	No data	OKU-NGS	360	331,60x	>40x for 99.81%, in the range of 1-40x for 0.06%, 0x for 0.13% of coding region sequences	2500.76x	>200x for 100.00%
**F2/P7**	Genomed	NimbleGen SeqCap EZ (Roche)	NextSeq 500 (Illumina)	OKU-NGS	318	387.45x	>40 for 98.00%, in the range of 1-40x for 0.02%, 0x for 1.98% of coding region sequences	-	-
**F3/P10**	Genomed	Twist Bioscience Custom Panel (Twist Bioscience)	No data	OKU-NGS	367	216x	>40x for 99.63%, in the range of 1-40x for 0.16%, 0x for 0.20% of coding region sequences	3732.95x	>200 for 100.00%
**F4/P15**	Genomed	Twist Bioscience Custom Panel (Twist Bioscience)	No data	OKU-NGS	360	270.60x	>40x for 99.75%, in the range of 1-40x for 0.07%, 0x for 0.18% of coding region sequences	4337.74x	>200x for 100.00%
**F5/P20**	Genomed	Twist Bioscience Custom Panel (Twist Bioscience)	No data	OKU-NGS	367	208.27x	>40x for 99.71%, in the range of 1-40x for 0.11%, 0x for 0.18% of coding region sequences	2605.53x	>200x for 100.00%

**F6/P23**	Genomed	Twist Bioscience Custom Panel (Twist Bioscience)	No data	OKU-NGS	317	239.83x	>40x for 99.87%, in the range of 1-40x for 0.04%, 0x for 0.10% of coding region sequences	-	-
**F7/P27**	Genomed	Twist Bioscience Custom Panel (Twist Bioscience)	No data	OKU-NGS	367	239.79x	>40x for 99.67%, in the range of 1-40x for 0.14%, 0x dla 0.19% of coding region sequences	2993.30x	>200 for 100.00%
**F8/P31**	Genomed	NimbleGen SeqCap EZ (Roche)	NextSeq 500 Illumina	OKU-NGS	274	261.12x	>40x for 99.44%, in the range of 1-40x for 0.53%, 0x for 0.03% of coding region sequences	-	-
**F9/P36**	Genomed	Twist Bioscience Custom Panel (Twist Bioscience)	No data	OKU-NGS	360	299.66x	>40 for 99.74%, in the range of 1-40 for 0.05%, 0x for 0.21% of coding region sequences	3048.68x	>200 for 100%
**F10/P39**	Genomed	NimbleGen SeqCap EZ (Roche)	NextSeq 500 Illumina	OKU-NGS	274	294.01x	>40 for 99.40%, in the range of 1-40x for 0.57%, 0x for 0.03% of coding region sequences	-	-
**F11/P46**	Genomed	Twist Exome 2.0 plus Comprehensive Exome Spike-In (Twist Bioscience)	No data	EXOME-1	20637	102.97x	>40 for 97.31%, in the range of 1-40x for 1.48%, 0x for 1.21% of coding region sequences	-	-
**F12/P47**	Genomed	Twist Bioscience Custom Panel	Element Biosciense Technology,	OKU-NGS	367	244.35x	>40x for 99.58%, in the range of 1-40x for 0.24%,	3432.61x	>200 for 100%

		(Twist Bioscience)	AVITI Platform				0x for 0.18% of coding region sequences		
**F13/P48**	Genomed	Twist Bioscience Custom Panel (Twist Bioscience)	Element Biosciense Technology, AVITI Platform	OKU-NGS	367	238.70x	>40x for 99.54%, in the range of 1-40x for 0.26%, 0x for 0.20% of coding region sequences	2070.22x	>200 for 100%
**F14/P49**	Asper Biogene	No data	No data	Congenital Stationary Night Blindness NGS panel	13	No data	No data	-	-
**F15/P50**	Genomed	Twist Bioscience Custom Panel (Twist Bioscience)	No data	OKU-NGS	360	186.38x	>40x for 99.53%, in the range of 1-40x for 0.27%, 0x for 0.21% of coding region sequences	2352.51x	>200x for 100%
**F16/P51**	Asper Biogene	No data	No data	Congenital Stationary Night Blindness NGS panel	13	No data	No data	-	-
**F17/P52**	Genomed	NimbleGen SeqCap EZ (Roche)	NextSeq 500 Illumina	OKU-NGS	274	248.61x	>40x for 99.38%, in the range of 1-40x for 0.60%, 0x for 0.02% of coding region sequences	-	-
**F18/P54**	Genomed	NimbleGen SeqCap EZ (Roche)	NextSeq 500 Illumina	OKU-NGS	318	244.54x	>40x for 97.89%, in the range of 1-40x for 0.13%, 0x for 1.98% of coding region sequences	-	-
**F19/P55**	Genomed	Twist Bioscience	Element Biosciense	OKU-NGS	367	241.13x	>40x for 99.60%, in the range of 1-40x for 0.21%,	3308.24x	>200x for 100%

		Custom Panel (Twist Bioscience)	Technology, AVITI Platform				0x for 0.18% of coding region sequences		
**F20/P56**	Genomed	Twist Bioscience Custom Panel (Twist Bioscience)	Element Biosciense Technology, AVITI Platform	OKU-NGS	367	201.66x	>40x for 99.48%, in the range of 1-40x for 0.34%, 0x for 0.18% of coding region sequences	1886.68x	>200x for 100%
**F21/P57**	Genomed	Twist Bioscience Custom Panel (Twist Bioscience)	Element Biosciense Technology, AVITI Platform	OKU-NGS	367	252,06x	>40x for 99.51%, in the range of 1-40x for 0.26%, 0x for 0.23% of coding region sequences	2291.72x	>200x for 100%
